# Burden of hypertension and associated factors among HIV-positive adults in Busia County, Kenya

**DOI:** 10.11604/pamj.2022.43.143.36394

**Published:** 2022-11-17

**Authors:** Ibrahim Oyawa, Maureen Adhiambo, Benard Wesonga, Maximilla Wanzala, Ferdinard Adungo, Olipher Makwaga, Matilu Mwau

**Affiliations:** 1 Department of Biomedical Sciences, Masinde Muliro University of Science and Technology, Kakamega, Kenya,; 2 Centre for Infectious and Parasitic Diseases Control Research, Kenya Medical Research Institute, Busia, Kenya

**Keywords:** Hypertension, HIV, adults, Busia County

## Abstract

**Introduction:**

the use of antiretroviral (ARVs) for the management of HIV (human immunodeficiency virus) infection has resulted in a prolonged lifespan among HIV-positive individuals. Both HIV infection and ARVs treatment put this population at a greater risk of developing hypertension. The study aimed at establishing the burden of hypertension and associated factors among HIV-positive population.

**Methods:**

a cross-sectional design was employed where a total of 280 HIV-positive adults in Busia County were selected in a multi-stage sampling procedure between March and August 2020. Sociodemographic, economic and behavioral information was collected using a structured questionnaire. Anthropometric measurements were taken using standard methods while clinical data were extracted from patients´ medical records. Proportion was used to establish hypertension burden. Analyses were done using the T-test, Chi-square, and odds ratio.

**Results:**

among the 280 study participants, 194 (69.3%) were females, and 239(85.4%) over 35 years of age. Hypertensive cases were 55 (19.6%). The hypertensive group had a significantly higher mean age (52.25±10.4 vs 44.9±11.3; p=0.002), waist-to-hip ratio (0.93±0.09 vs 0.89±0.07; p=0.016), HIV duration (8.64±4.63 vs 6.86±4.04; p=0.014) and cumulative ART treatment duration (8.31±4.61 vs 6.68±3.93; p=0.018). Factors found to be significantly associated with hypertension in the bivariate analysis included age (p=0.003); family history (p=0.024); duration of alcohol intake (p=0.034); HIV duration (p=0.033) and treatment duration (p=0.043). In the multivariate analysis, only age (p=0.045) and family history (p=0.018) contributed significantly in the logistic regression model.

**Conclusion:**

the study revealed a slightly lower burden of hypertension among HIV -positive adults in Busia County. Age and family history were the factors independently associated with hypertension in this study.

## Introduction

The use of highly active antiretroviral therapy (HAART), a combination of three antiretroviral drugs for the management of HIV infection, has transformed the disease from a rapidly lethal illness into a chronic condition. With a global antiretroviral therapy coverage of about 15.8 million and around 12 million people living with HIV in sub-Saharan Africa, the survival rate has dramatically increased [1-3]. In Kenya, about 1.5 million (4.9%) people are living with HIV, with ART coverage of close to 80% [4]. As of 2017, the preferred first-line regimens for adults were TDF+3TC+EFV, TDF+3TC+NVP, AZT+3TC+EFV, or AZT+3TC+NVP while the second line included AZT+3TC+LPV/r, AZT+3TC+ATV/r, TDF+3TC+LPV/r or TDF+3TC+ATV/r [5], as shown in Table 1. In 2018, these guidelines were reviewed to include dolutegravir (DTG) in the first-line combination [6]. Currently, the commonly used regimens include ABC/TDF+3TC+DTG, TDF+3TC+EFV, AZT/TDF+3TC+ATV/r and ABC+3TC+LPV/r with majority of HIV-positive individuals being on TDF+3TC+DTG [7]. As the lifespan of people on ART increases, challenges of long-term HIV infection and toxic effects of ART emerge [8]. Cardiovascular diseases (CVD) are among the challenges that occupy a prominent position as a major source of morbidity and mortality in HIV-infected patients [9]. This has necessitated a paradigm shift of treatment focus towards the long-term management of HIV infection and chronic complications associated with ART usage. Hypertension confers the highest attributable risk of CVD occurrence and accounts for 7.6 (13.5%) million deaths per annum worldwide [10]. Globally, around 22% (1.13 billion) of the adult population had hypertension by the year 2015 [11] with prevalence ranging from 6 to 48% in sub-Saharan Africa [12]. In Kenya, the prevalence was found to be 24.5% as per the Kenya stepwise survey for non-communicable diseases (NCD), 2015 Report [13]. Both ART, especially protease inhibitors (PIs), and HIV itself can cause lipodystrophy and dyslipidemia that may cause hypertension through a simultaneous accumulation of central adiposity and atrophy of peripheral adiposity [14]. In addition to HIV infection and ART usage, certain demographic characteristics, socio-economic and behavioral risk factors for hypertension found in the general population also occur among people living with HIV. They include the family history of hypertension and diabetes, age, male gender, smoking, physical activity, diet, alcohol, obesity, Diabetes mellitus and hyperlipidemia [15]. This exposes HIV-positive individuals to a greater risk of developing hypertension than the general population. Despite this, few studies have been done with varying reports on the burden. A recent meta-analysis of global data demonstrated that 35% of all HIV-positive adults on ART have hypertension, compared with an estimated 30% of HIV-negative adults [16] while in a hospital-based cross-sectional study done in Spain, a prevalence of 20.6% was reported, which was lower than the global value [15].

**Table 1:** classification of ART regimens

Regimen	Codes	Market name
First line	TDF+3TC+EFV	Tenofovir+lamivudine+efavirenz
	TDF+3TC+NVP	Tenofovir+lamivudine+nevirapine
	AZT+3TC+EFV	Zidovudine+lamivudine+ efavirenz
	AZT+3TC+NVP	Zidovudine+lamivudine+ nevirapine
Second line	AZT+3TC+LPV/r	Zidovudine+lamivudine+ritonavir boosted lopinavir
	AZT+3TC+ATV/r	Zidovudine+lamivudine+ritonavir boosted atazanavir
	TDF+3TC+LPV/r	Tenofovir+lamivudine+ritonavir boosted lopinavir
	TDF+3TC+ATV/r	Tenofovir+lamivudine+ritonavir boosted atazanavir

In Ethiopia, a study conducted found a prevalence of 53% among HIV positive population, which was much higher compared to 20% found in the general population [17] while in Uganda, a prevalence of 22.6% was reported in a study performed to assess the prevalence of cardio-metabolic risks among HIV positive adults [18]. In another cohort study carried out in Tanzania to determine incidence and risk factors for hypertension among HIV patients in rural populations, a prevalence of 11.6% was found among ART naïve patients at the recruitment stage and an incidence of 9.6% during a follow-up [19]. In Kenya, few studies have been carried out to estimate the prevalence of hypertension in HIV-positive patients. In one study conducted among HIV- infected individuals visiting Kenyatta National Hospital, an overall prevalence of 13.6% was reported with 12.9% in the HAART experienced and 14.3% in the HAART naive group [20]. In another study done among the same population attending Thika district hospital, a prevalence of 18% was found [21]. A more recent study performed among HIV- positive clients visiting Kenyatta National Hospital reported a prevalence of 23.2% [22]. This indicates that the burden of hypertension may be increasing with time and therefore the need for continuous monitoring of the trends. With the evidence linking HIV infection with hypertension, there is fear that areas with high HIV prevalence could also be having a high prevalence of hypertension. Busia is among the top five counties in Kenya with high HIV Prevalence [23] yet to the best of our knowledge, no published study has been done to establish the burden of hypertension and associated risk factors among HIV-positive adults. The few studies which have been done in Kenya have concentrated on the prevalence within limited study sites and majorly among the urban population. This study aimed at determining the burden of hypertension among HIV-positive populations living in the rural settings of Kenya and from more than one study sites. It also aimed at establishing the socio-demographic, economic, behavioral, clinical and anthropometric measures associated with the observed burden.

## Methods

**Study design:** this was a cross-sectional study carried out over six month´s period using WHO STEPwise approach to Surveillance of non-communicable diseases [24]. Participants were classified as either hypertensive or non-hypertensive based on repeated blood pressure measurements and whether one is on hypertensive medication or not. The design was chosen due to its suitability of determining point prevalence and in establishing association between risk factors such as age with outcome variable which was hypertension.

**Figure 1:**
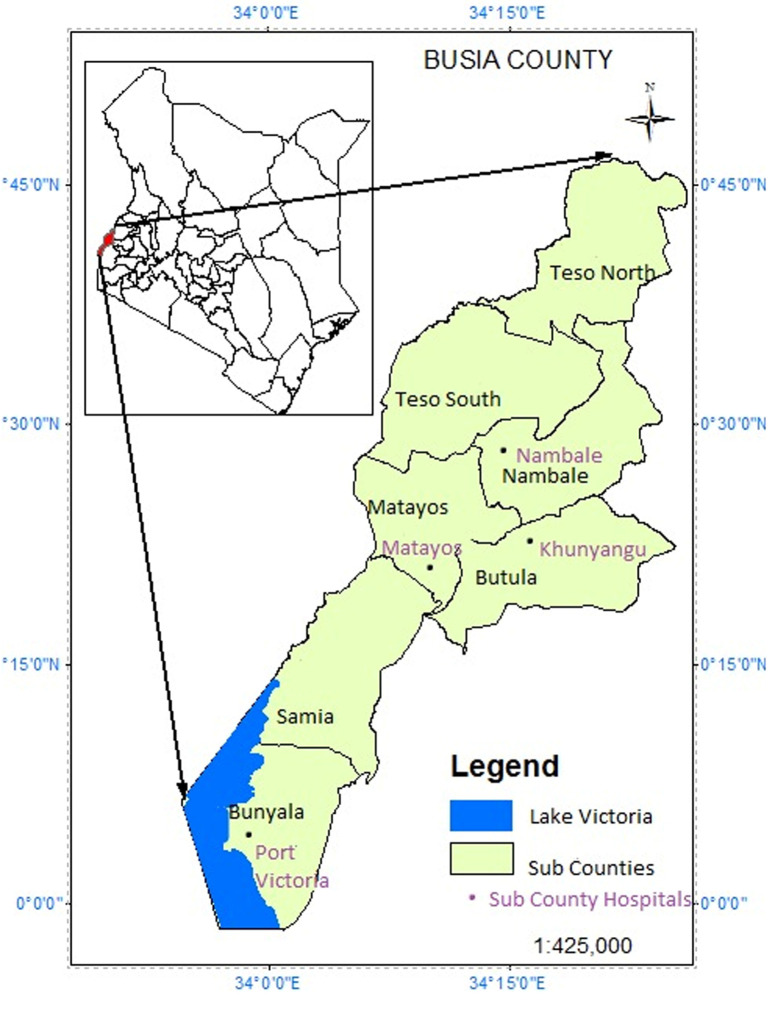
study sites in Busia County, Western Kenya

**Study setting:** the study was conducted in four sub counties of Busia County, Western Kenya (Figure 1). The county is among the top five counties in Kenya with high HIV prevalence rate [23]. Adult HIV prevalence stands at 6.7% (approximately 26,590) with ART coverage of 92% [23]. Busia County has seven sub-counties namely Bunyala, Butula, Nambale, Teso North, Teso South, Matayos and Funyula. According to available statistics, Bunyala Sub-county leads in HIV prevalence with 8.2%. Samia/Funyula is second with 7.4% and Butula third at 6.3%. Teso North is placed fourth at 4.2%. Matayos and Teso South tie on fifth place at 3.2% with Nambale having the least prevalence of 3.1% [25]. Four Sub-counties were purposively selected for this study. Study participants were drawn from comprehensive care centres of the Sub-county hospitals. The sub-county hospitals included Port Victoria, Khunyangu, Nambale and Matayos. Busia County has a population of 893,681 with 467,401 (52.3%) females, 426,252 (47.7%) males and 28 intersexes. Majority (87%) of its population live in the rural set-ups with 44.4% (396,794) of the population falling between age group 18 years and above [26]. The major economic activities include subsistence agriculture, fishing and small-scale commerce. The county has a unemployment rate of 66.7% and illiteracy level 43.3% against the national level of 47.2%, and 33.6% respectively [26,27].

**Study population:** the study population were HIV-positive adults on ARTs attending the comprehensive care center (CCC) of the selected Sub-county hospitals (Port Victoria, Khunyangu, Nambale and Matayos) and who agreed to participate. Patients who were clinically ill and pregnant women (as visually observed by the caregiver) were excluded from the study.

**Sampling method:** multistage sampling was used. The first stage involved purposive sampling of the four out of the seven sub-counties based on their HIV prevalence. The second stage involved non-proportionate convenient sampling of study participants from the four selected sub-county hospitals. This involved recruiting participants who met the inclusion criteria until the required number of 70 was reached for each hospital. A total of 280 participants were selected for the study over a period of six months (March, 2020 to August, 2020) with each sub-county hospital contributing equally to the number. The use of multistage sampling was employed to minimize the effect of selection bias.

**Data collection procedures:** sociodemographic and economic (age, sex, marital status, family history of hypertension, education level, occupation, income level), lifestyle characteristics (tobacco use, alcohol consumption, diet, physical activity) and some clinical information (duration of HIV infection, history of kidney disease, history of diabetes) were collected using semi-structured questionnaire. Other clinical data (type of ARVS and duration of treatment) were obtained from the patients´ health records. Trained nurses and clinicians were selected in each hospital and additionally trained on the questionnaire. Physical measurements (height, weight, waist and hip circumference) were taken using standard methods and recorded on the questionnaire. Height was measured in meters using a height rule while weight was measured in kilograms using a standard weighing machine. These two measurements were used to calculate body mass index (BMI). Both waist and hip circumference were taken in centimeters. The waist-hip ratio (WHR) was calculated as a ratio of the waist circumference to the hip circumference. Blood pressure measurements were taken non-invasively using a mercury column sphygmomanometer (first and fifth phases of Korotkoff sounds taken as SBP and DBP respectively) after the participants have rested for 5 minutes in sitting position. The second measurement was taken after five minutes and the average of the two readings calculated. In this study, hypertension was defined as having an average systolic blood pressure (SBP) of ≥140 and/or a diastolic blood pressure (DBP) ≥90 mmHg or both; being previously diagnosed with hypertension by any health professional or taking antihypertensive medication [28]. Prehypertension was defined as having an average SBP of between 120-139mmHg and/or an average DBP of between 80-89mmHg or both. An individual was considered normotensive if the average SBP was less than 120mmHg and DBP less than 80mmHg, not having been previously diagnosed with hypertension and not under any antihypertensive medication.

**Sample size determination:** with a prevalence rate of 23%, a deviation of ±5% in the estimated prevalence and a confidence interval (CI) of 95%, the sample size of 280 was arrived at using Fischer formula [29].


$$N_{o}=\frac{Z^{2}p(1-p)}{d^{2}}$$

Where N_0_ is the minimum sample size required, Z the z-score (1.96) at 95% CI, P is the estimated prevalence of hypertension (23), d is the standard deviation from the estimated prevalence (±5%). The 23% prevalence used was obtained from a published study which was done at Kenyatta National Hospital in the year 2015 with 297 HIV positive.

**Statistical analysis:** data was entered, cleaned and stored into the computer using SPSS statistical software version 20.0. Analysis was done using the latest version of SPSS [30]. Missing data were excluded from the analysis of variables with incomplete information. Percentages, means, and frequencies were used to summarize the data which was then presented in figures and tables. The study participants were grouped as either hypertensives or non-hypertensives depending on their blood pressure measurements and whether they are on antihypertensive medication. Proportions was used to estimate the burden of hypertension in the population while the association between risk factors and hypertension was assessed using Chi-square and odds ratio at 95% confidence intervals. T-test was used to check if there were statistical differences among continuous variables between the two groups of outcome variable. Logistic regression was used to develop the final model for factors found to have a p-value ≤0.25 and with a sample size of more than 250. All tests were 2-tailed and P<0.05 was considered statistically significant.

**Ethical considerations:** ethical approval was sought from Masinde Muliro University of Science and Technology Ethical Review Committee (MMUST/IERC/104/19). Permission to conduct the study was obtained from National Council of Science and Technology (License No: NACOSTI/P/20/3694) and from Busia County Health Office (CG/BSA/ADM/1/56/VOL.11/58/7).

## Results

**Sociodemographic and economic characteristics of the study population:** there were 280 participants drawn from 4 sub-county hospitals of Nambale, Matayos, Port Victoria and Khunyangu. Out of this,194 (69.3%) were females. The mean age was 46.35 (SD±11.52) with a minimum age of 21 years and a maximum age of 80 years. Median age was 45 years (IQR: 30-60) with majority (85.4%) falling within age group 35 years and above. Participants with no basic education were 55 (19.6%), 154 (55%) were married and 251 (89.6%) were unemployed. A majority (80.4%) were living below the poverty level of less than 60 dollars per month (Table 2). Those who had a history of hypertension were 65 (23.3%) and, 20 (7.2%) could not recall any history of hypertension in their families. Out of the 280 study participants, 148 (52.9%) had their waist and hip circumference measured while 132 (47.1%) objected the measurements due to SARs-CoV-2 fears. Only 119 (42.5%) of the study population were normotensive. The remaining 161 (57.5%) were either in pre-hypertensive or hypertensive stage. Among the non-normotensive, 106 (37.9%) were on pre-hypertensive stage while 55 (19.6%) were hypertensive, giving a point prevalence of 19.6%. Out of the 55 hypertensive cases, 17 (30.9%) were on antihypertensive medication with 5(29.4%) having their blood pressure controlled.

**Table 2:** sociodemographic and economic factors associated with hypertension among HIV-positive adults in Busia County

N=280	Frequencies	Hypertensive group n=55 (19.6%)	Non hypertensive n=225 (80.4%)	P-value
**Age (in years)**		Mean;52.25; SD ±10.40	Mean;44.9; SD ±11.30	0.002a
Under 35	41(14.6%)	2(4.9)	39(95.1)	
35 yrs and above	239(85.4%)	53 (22.2)	186 (77.8)	0.003*
**Gender**				
Male	86(30.7%)	21(24.4)	65(76.5)	
Female	194(69.3%)	34(17.5)	160(82.5)	0.176
**Sub-county**				
Bunyala	70(25%)	15(21.4)	55(78.6)	
Butula	70(25%)	15(21.4)	55(78.6)	
Matayos	70(25%)	12(17.1)	58(82.9)	
Nambale	70(25%)	13(18.6)	57(81.4)	0.894
**Marital Status**				
Single	13(4.5%)	1(7.7)	12(92.3)	
Married	154(55%)	29(18.8)	125(81.2)	
Separated/Divorced	26(9.3%)	5(19.2)	21(80.8)	
Widowed	87(31.2%)	20(23)	67(77)	0.602
**Level of education**				
None	55(19.6%)	13(23.6)	42(76.4)	
Primary	168(60%)	28(16.7)	140(83.3)	
Secondary	47(16.8%)	11(23.4)	36(76.6)	
College/University	10(3.6%)	3(30)	7(70)	0.459
**Employment status**				
Employed	29(10.4%)	6(20.7)	23 (79.3)	
Unemployed	251(89.6%)	49(19.5)	202 (80.4)	0.876
**Monthly Income level (dollars))**				
Less than 60	225(80.4%)	44(19.6)	181 (80.4)	
More than 60	55(19.6%)	11(20)	44 (80)	0.940
**FH of hypertension, N=260**		**n=51**	**n=209**	
Yes	65(23.4%)	19(29.2)	46 (70.8)	
No	195(69.6%)	32(16.4)	163 (83.6)	0.029*

Chi-square test (χ2) for categorical variables; *Statistically significant, p≤0.05 T-test for continuous variables; a Statistically significant; p≤0.05 FH: Family history; N:overall sample size; n:sample size in the group

**Mean differences between groups:** a student´s T-test revealed mean differences between hypertensive and non-hypertensive groups to be statistically significant (Table 3) in terms of age (52.25±10.4 vs 44.9±11.3; p-value=0.002), HIV duration (8.64±4.63 vs 6.86±4.04; p-value =0.014), ART duration (8.31±4.61 vs 6.68±3.93; p-value=0.018) and WHR (0.93±0.09 vs 0.89±0.07; p-value=0.016) but not with BMI (21.78±4.00 vs 22.00±4.32; p-value =0.710). The means were significantly higher among the hypertensive than the normotensive groups.

**Table 3:** behavioural factors associated with hypertension among HIV-positive adults in Busia County

N=280	Hypertensive group, n=55 (19.6%)	Non hypertensive, n=225 (80.4%)	P-value
**Diet**			
**Frequency of taking fruits and vegetables (days/week)**			
<4 days per week	11(16.4)	56(83.6)	
>4 days per week	44 (20.7)	169 (79.3)	0.453
**Milk**			
Yes	42 (18.5)	185 (81.5)	
No	13 (24.5)	40 (75.5)	0.317
**Salt**			
Yes	21 (19.6)	86 (80.4)	
No	34 (19.7)	139 (80.3)	0.986
**Processed flour**			
Yes	9(19.6)	37 (80.4)	
No	46(19.7)	188 (80.3)	0.982
**Physical activity**			
No	42(20.6)	162(79.4)	
Yes	13(17.1)	63(82.9)	0.506
**Frequency of physical activity, N=76**	**n=13**	**n=63**	
<4 times per week	8 (16.0)	42 (84.0)	
>4 times per week	5 (19.2)	21 (80.8)	0.721
**Smoking**	8 (25)	24 (75)	
Smoker			
Never smoked	47(19)	201(81)	0.42
Duration of smoking(years), **N=32**	**n=8**	**n=24**	
>5 yrs	5 (31.2)	11 (68.8)	
< 5 yrs	3(18.8)	13 (81.2)	0.409
**No of sticks smoked per day, n=32**			
<5 sticks per day	3 (18.8)	13 (81.2)	
>5 sticks per day	5 (31.2)	11 (68.8)	0.413
**Alcohol intake**			
Drinker	22 (20.4)	86 (79.6)	
Never drunk	33(19.2)	139(80.8)	0.805
**Duration of drinking (years), N=108**	**n=22**	**n=86**	
>5 yrs	14 (29.8)	33 (70.2)	
< 5 yrs	8 (13.1)	53 (86.9)	0.034*
**No of bottles taken/day, N=108**	**n=22**	**n=86**	
<5 bottles per day	18 (20)	72 (80)	
>5 bottles per day	4 (22.2)	14 (77.8)	0.758

**Sociodemographic and economic factors:** age was found to be significantly associated with hypertension with those above 35 years being 5.56 (95% CI: 1.30-23.77) times more likely to be hypertensive as compared to those below 35 years old (p-value =0.003). Among those who had a family history of hypertension, 19 (29.2%) were hypertensive compared to 32 (16.4%) of those who did not have it. This association was found to be significant with those having the history being 2.10 (95% CI: 1.09-4.05) times more likely to be hypertensive (p-value=0.029). Gender, sub-county, marital status, level of education, employment status and income level did not show any significant association with hypertension (p-value>0.05), as shown in Table 4.

**Table 4:** clinical and anthropometric measures associated with hypertension among adult HIV-positive population in Busia County

N=280	Hypertensive group, n=55 (19.6%)	Non hypertensive, n=225 (80.4%)	χ2 P-value
**HIV duration**	Mean;8.64; SD±4.63	Mean;6.86; SD ± 4.04	0.014*
9 years and above	31(25.6)	90 (74.4)	
Less than 9 years	24(15.1)	135 (84.9)	0.033*
**Cumulative ART duration**	Mean;8.31; SD ±4.61	Mean;6.68; SD ±3.93	0.018*
9 years and above	29(25.4)	85 (74.6)	
Less than 9 years	26(15.7)	140 (84.3)	0.043*
**Type of ARVs regimens**			
Ritonavir boosted	9 (20.5)	35 (79.5)	
EFV/NVP/DTG containing	46 (19.5)	190 (80.5)	0.883
**History of kidney disease**			
Yes	1(25)	3(75)	
No	54(19.6)	222(80.4)	0.786
**History of diabetes**			
Yes	1(20)	4(80.4)	
No	54(19.6)	221(80.4)	0.982
**Anthropometric measures**			
BMI Cut-offs	Mean 21.78; SD±4.00	Mean;22.00; SD±4.32	0.71
Below 18.5	9(17.3)	43(82.7)	
18.5-24.9	35(19.9)	141(80.1)	
25-29.9	7(18.9)	30(81.1)	
30 and above	4(26.7)	11(73.3)	0.875
**WHR, N=148**	Mean;0.93; SD±0.09**n=28**	Mean;0.89; SD±0.07**n=125**	0.016*
0.9 and below	11(12.4)	78(87.6)	
Above 0.9	12(20.3)	47(79.7)	0.194

**Behavioral factors associated with hypertension:** among the behavioral characteristics, only duration of alcohol intake was found to be significantly associated with hypertension with those who have drunk for more than 5 years being 2.81(95% CI: 1.06-7.42) times more likely to be hypertensive compared to those who have drunk for less than 5 years (p-value=0.034). Other behavioral factors such as diet, physical activity and smoking did not show a significant association with hypertension (p>0.05) even though a slightly higher percentage of hypertensives were observed among smokers, those who don´t take milk and those who are physically inactive (Table 5).

**Table 5:** bivariate and multivariate analysis of factors associated with hypertension among HIV-positive adults in Busia County

	Hypertensive, n=55(19.6%)	Non-hypertensive, n=225 (80.4%)	Bivariate, OR (95% CI)	P-value	Multivariate ,OR(95%CI)	P-value
**Age (in years)**						
Under 35 yrs	2(4.9)	39(95.1)				
35 yrs and above	53 (22.2)	186 (77.8)	5.56(1.30-23.77)	0.003*	4.9(1.09-21.8)	0.037*
**Gender**						
Male	21(24.4)	65(76.5)				
Female	34(17.5)	160(82.5)	1.52(0.82-2.81)	0.182	0.56(0.29-1.06)	0.075
**FH of hypertension, n=260**						
Yes	19(29.2)	46 (70.8)				
No	32(16.4)	163 (83.6)	2.10(1.09-4.05)	0.029*	2.39(1.21-4.73)	0.012*
**Duration of alcohol intake, n=108**						
>5 yrs	14 (29.8)	33(70.2)				
< 5 yrs	8 (13.1)	53 (86.9)	2.81 (1.06-7.42)	0.034*		
**HIV duration**						
9 years and above	31(25.6)	90(74.4)				
Less than 9 years	24(15.1)	135(84.9)	1.94(1.07-3.52)	0.033*	1.83(0.31-10.6)	0.504
**Cumulative ART duration**						
9 years and above	29(25.4)	85(74.6)				
Less than 9 years	26(15.7)	140(84.3)	1.84(1.01-3.33)	0.043*	0.93(0.16-5.38)	0.939
**WHR, n=148**						
0.9 and below	11(12.4)	78(87.6)				
Above 0.9	12(20.3)	47(79.7)		0.194		

**Clinical factors and anthropometric measures:** there was a significant association between HIV duration and hypertension. Those who have been HIV positive for more than 9 years were 1.94 (95% CI: 1.07-3.52) times more likely to be hypertensive compared to those who have been positive for less than 9 years (p-value =0.033). All participants were on ART with 84.3% (236) being on non-nucleotide reverse transcriptase inhibitor combinations (2NRTIs+EFV or NVP or DTG) and 15.7% (44) on ritonavir boosted Protease inhibitors (PI) combinations (2 NRTIs+ATV/r or 2 NRTIs+LPV/r). There was no significant association between ART type and hypertension (p-value>0.05). However, the cumulative duration of ART intake had a significant association with hypertension. Those who have been on ART for more than 9 years were 1.84 (95% CI: 1.01-3.33) times more likely to be hypertensive compared to those who have taken ARVs for a period less than 9 years (p-value =0.043). History of diabetes, kidney disease, BMI and WHR did not show any signs associated with hypertension (p>0.05) although a higher percentage of hypertensives (20.3% vs 12.4%) had waist to hip ratio of above 0.9 (Table 5). Five factors were found to be significantly associated with hypertension in bivariate analysis. These were; Age (OR: 5.56; 95% CI: 1.30-23.77; p-value=0.003), Family history (OR: 2.10; 95% CI: 1.09-4.05; p-value=0.029), duration of alcohol intake (OR: 2.81; 95% CI: 1.06-7.42; p-value=0.034), HIV duration (OR: 1.94 ;95% CI: 1.07-3.52; p-value =0.033) and cumulative ART duration (OR: 1.84; 95% CI: 1.01-3.33; p-value =0.043). In the multivariate logistic analysis, only age (OR: 4.9; 95% CI: 1.09-21.8; p=0.037) and family history (OR: 2.39; 95% CI: 1.21-4.73; p=0.012) were found to significantly contribute in the logistic regression model. Duration of alcohol intake, gender, HIV infection and ART treatment duration were the confounders adjusted for in the multivariate analysis. They were included in this analysis since they had a p-value ≤ 0.25 in the bivariate analysis.

## Discussion

From this study, the prevalence of hypertension was found to be 19.6% among HIV positive adult population in Busia County. This was slightly lower than the 24.5% prevalence obtained from the 2015 Kenya STEPs survey of the general population [31] and 23.2% recently reported among people living with HIV in the urban settings of Kenya [22]. The prevalence was also lower than the 35% reported globally [16], 53% in Ethiopia [17], 28.7% found in Tanzania [32] and almost equals to 22.6% found in Uganda [18]. This variation could be due to the differences in the geographical locations or setting from where the study participants were drawn and from the study population itself. For example, the prevalence tends to be higher among urban dwellers than in rural settings even within the same population of HIV positive individuals [33,34]. Increased exposure to behavioural risk factors such as sedentary lifestyle and processed food among the urban population could be driving the high burden [35]. The prevalence also appears to be lower among HIV positive individuals than the general population. Increased access to healthcare services and interaction with prevention messaging regarding risk factors for hypertension could be behind such observation. Our current prevalence was higher than the 13.6% and 18% reported in 2009 and 2014 respectively [20,21] and differences in time series could better explain this. In Busia County, slight variation was also observed across different sub-counties with those having high HIV prevalence also recording high hypertension prevalence. This suggests that HIV could be driving hypertension burden. However, the variation was not statistically significant (p>0.05).

Among demographics and socio-economic characteristics, age and family history were found to be positively associated with hypertension, concurring with other studies conducted in Kenya and Spain [21,36]. While an age of 40 years and above was found to be significantly associated with hypertension in the general population [37], this study found an age of above 35 years to be a predictor of hypertension, suggesting that HIV persons on HAART could be developing hypertension at an earlier age. Our results indicate that adults with a family history of hypertension were 2 times more likely to be hypertensive than adults with no such history. Many other studies have reported the existence of such an association [18,32,36]. Genetic heritability of factors associated with hypertension such as elevated serum cholesterol and family sharing of cultural/environmental factors could be the link through which this association occurs. We did not find any significant association between hypertension and gender, marital status, employment status, education level or income level. Similar findings have been reported by many researchers [21,38-40]. However, few studies conducted in India and Korea reported male gender, being married, unemployment, low education and low income level to be significant risk factors for hypertension [41,42]. Even though gender was not statistically associated with hypertension in this study, higher odds of hypertension was witnessed among males. A higher percentage of males than females are believed to be involved in behavioural risk factors such as smoking and alcohol intake which predispose them to hypertension. The variations observed in the relationship between other sociocultural and economic indicators with hypertension highlight the complexities of understanding the influence of social status on long term health outcomes. A more refined study design such as longitudinal is necessary to link education status with employment and income level and to see how these relate with hypertension occurrences.

Dietary intake of salt, milk, processed flour or the frequency of intake did not significantly associate with hypertension. This disagreed with many researchers who have reported poor dietary habits such as excessive consumption of raw salt, processed foods and being non-vegetarian to be risk factors for the development of hypertension [43-45]. Fruits and vegetables are believed to have a protective effect against hypertension due to their high content of potassium, magnesium and fibres. The fact that all the participants in this study reported to have been taking fruits and vegetables hindered the establishment of this association with hypertension. Despite the lack of association between milk and hypertension in our study, a lower percentage of participants who consume milk were hypertensive (18.5% vs 24.5%). Other clinical and animal studies have reported the antihypertensive effects of milk [46,47]. Neither physical activity nor the frequency was found to be significantly associated with hypertension in this study although those who were physically inactive had higher odds of being hypertensive (OR 1.26). However, several other studies have reported inverse association with sedentary lifestyle being a significant risk factor for hypertension and having engaged in physical activities for most days of the week to be beneficial association [48-50]. Regular physical activity is believed to strengthen heart muscles thereby lowering the blood pressure. Our study had a limitation in that it did not capture other forms of physical activities such as farming, which is common among the rural population where the study participants were drawn. Although there was no significant association between either alcohol intake or the number of bottles taken per day, duration of drinking was found to be significantly associated with hypertension (OR 2.81). Other studies have reported similar findings, but not with a duration of drinking [41-50]. Recent studies have shown that chronic ethanol ingestion induces hypertension supporting our findings [51,52]. While some studies reported alcohol to be a significant risk factor for hypertension [32,53], other researchers found alcohol to have protective effects [54,55]. These differences in findings might have occurred due to variations in the amount and concentration of alcohol consumed. Alcohol ingestion may raise blood pressure by decreasing the vasodilators such as nitric oxide in the vascular endothelium. This harmful effect should not support the presumed cardioprotective effect of low-to-moderate consumption of alcoholic drinks.

The association between smoking and hypertension have been controversial. While few studies have reported no association between the two [21,56], others have reported lower blood pressure levels among smokers than in non-smokers [57,58] while some researchers have found a strong association between the two [49,59,60]. In our study, higher odds of hypertension were found among smokers, those who have smoked for more than 5 years and those who smoked more than 5 sticks per day. However, these associations were not statistically significant. Smoking for a prolonged duration of time builds itself up to a threshold level, initiating vascular changes in blood vessels which ultimately results in hypertension. Duration of HIV infection and HAART treatment were the clinical factors found to be significantly associated with hypertension in this study. Other studies have reported similar findings with duration of HIV infection and ART use of more than 5 years to be increasing the likelihood of being hypertensive [61-63]. In contrast, some did not find any association [64-66]. We speculated that the shorter duration of HIV infection and ART treatment (less than 5 years) among their study participants could have contributed to the observed lack of association. HIV infection is believed to cause hypertension through chronic immune activation and subsequent immune suppression. Prolonged use of ART may result in lipodystrophy and dyslipidaemia that may cause hypertension through accumulation of central adiposity. There was no significant association between hypertension and ART type, history of diabetes or kidney disease. Even though PI based regimens have been linked with the development of hypertension in some studies [62,67], this study did not find such an association. Massive transitioning of patients from other regimens including PI based to DTG based regimens over the last 2 years might have biased the findings. Similarly, due to the low number of participants who had history of diabetes or kidney disease or both, we could not confidently debate for or against their association with hypertension. None of the anthropometric measures was found to be significantly associated with hypertension disagreeing with Njeru [21] who found BMI above 30 to increase the likelihood of being hypertensive. This could be due to the fact that majority of the study participants (84%) were not on PI based regimens which have been linked with changes in body fat distributions and increased waist-to-hip ratio. Nonetheless, higher BMI and WHR have been linked with the development of hypertension in many other studies [50,68,69] underscoring the importance of monitoring these measures among HIV positive individuals

**Study limitations:** this being a cross-sectional study, the causal relationship could not be deduced. Some patients might have been on different regimens over a period of time introducing a challenge of associating a particular ART regimen with an outcome. Most of the socio-economic and behavioral responses could not be confirmed beyond self-reporting hence possibility of response and recall biases.

## Conclusion

The study reveals the burden of hypertension among HIV positive adult population living in the rural settings of Busia County to be 19.6% which was slightly lower than the 23.2% recently reported in urban population of Nairobi County. Longer duration of HIV infection and ART treatment of ≥9yrs, prolonged alcohol intake of more than 5 years, age and family history are the factors found to be significantly associated with hypertension in this study. An older age of above 35 years and having a positive family history of hypertension were the factors independently associated with hypertension. We recommend continuous monitoring of trends in hypertension among people living with HIV as the duration of treatment becomes longer. There is also a need to find out specific ART options that are associated with hypertension for proper decision-making during treatment initiation or regimen change.

### What is known about this topic

- The burden of hypertension among the general population has been widely documented;
- There are known traditional risk factors that predispose the general public to hypertension; these traditional risk factors also exist among people living with HIV.

### What this study adds

- The study demonstrated that in additional to the common risk factors for hypertension, people living with HIV are also faced with additional factors such as HIV infection and treatment duration; this could put them at a greater risk of developing hypertension;
- The study also suggests that the burden of hypertension among the rural population is lower than that of the urban population;
- Our study provides a baseline data upon which future trends in the burden of hypertension among HIV positive population can be based.
